# Supplementary material for: Olefin oligomerization by main group Ga3+ and Zn2+ single site catalysts on SiO2
Source: Nat Commun. 2021 Apr 19;12:2322. doi: 10.1038/s41467-021-22512-6 (PMC8055657; doi:10.1038/s41467-021-22512-6)
Supplement: Supplementary file 2 — Supplementary Information [file 41467_2021_22512_MOESM2_ESM.pdf]

Supplementary Information for

Olefin Oligomerization by Main Group Ga^{3+} and Zn^{2+} Single Site Catalysts on SiO_2

Nicole J. LiBretto[a,§], Yinan Xu[a,§], Aubrey Quigley[a], Ethan Edwards[a], Rhea Nargund[a], Juan Carlos Vega-Vila[a], Richard Caulkins[a], Arunima Saxena[a], Rajamani Gounder[a], Jeffrey Greeley[a], Guanghui Zhang[a,b], Jeffrey T. Miller[a*]*

[a] Davidson School of Chemical Engineering, Purdue University, 480 Stadium Mall Drive, West Lafayette, Indiana 47907, United States

[b] State Key Laboratory of Fine Chemicals, PSU-DUT Joint Center for Energy Research, School of Chemical Engineering, Dalian University of Technology, Dalian, Liaoning 116024, PR China

[§] N.L. and Y.X. contributed equally.

Corresponding authors: [*] gzhang@dlut.edu.cn (G.Z.); mill1194@purdue.edu (J.T.M.)

Table of Contents

Supplementary Figures 3

Supplementary Tables 11

Supplementary Methods 19

Supplementary Notes 20

  Supplementary Note 1 20

  Supplementary Note 2 21

  Supplementary Note 3 21

  Supplementary Note 4 23

  Supplementary Note 5 23

  Supplementary Note 6 23

Supplementary Discussion 27

Supplementary References 28

Supplementary Figures

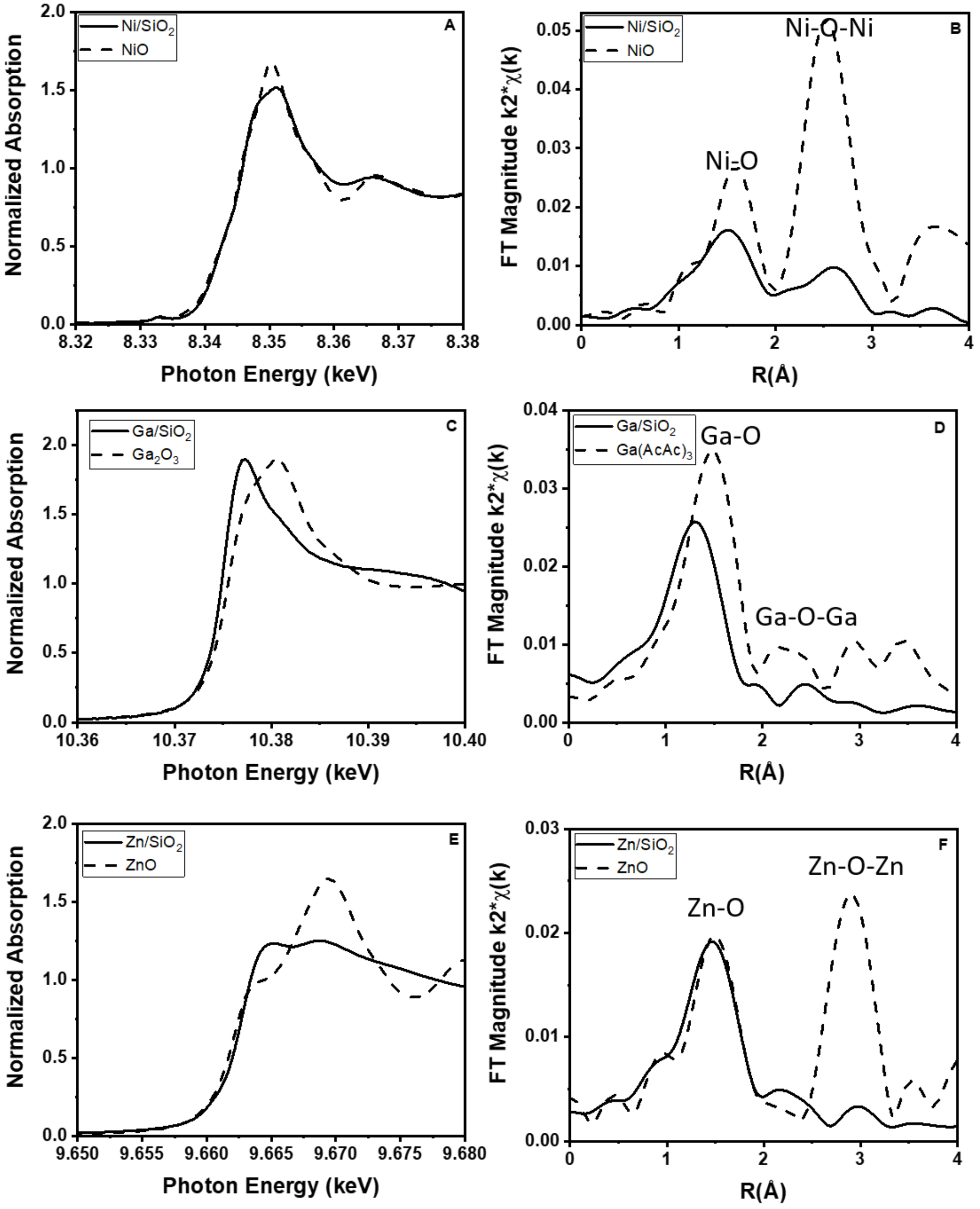

Supplementary Figure 1. XAS of dehydrated catalyst structures for Ni/SiO_2. a XANES of Ni/SiO_2 and NiO. **b** Fourier transform of the k^2-weighted EXAFS of Ni/SiO_2 and NiO. **c** XANES Ga/SiO_2 and Ga_2O_3. **d** Fourier transform of the k^2-weighted EXAFS of Ga/SiO_2 and Ga_2O_3. **e** XANES of Zn/SiO_2 and ZnO. **f** Fourier transform of the k^2-weighted EXAFS of Zn/SiO_2 and ZnO.

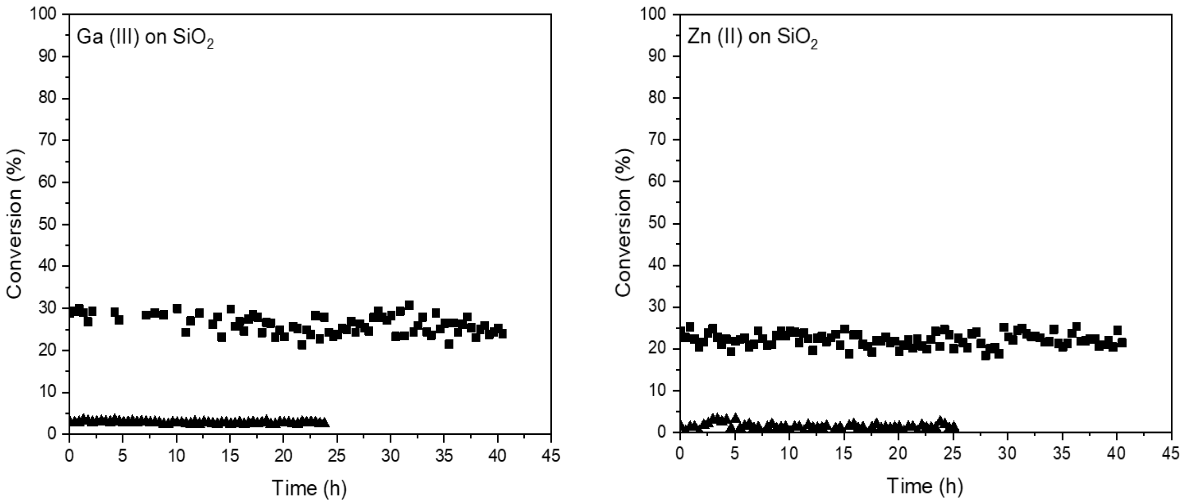

Supplementary Figure 2. **1 Atm ethylene oligomerization at 250°C (▲) and 450°C (■). a** Ga/SiO_2. **b** Zn/SiO_2.

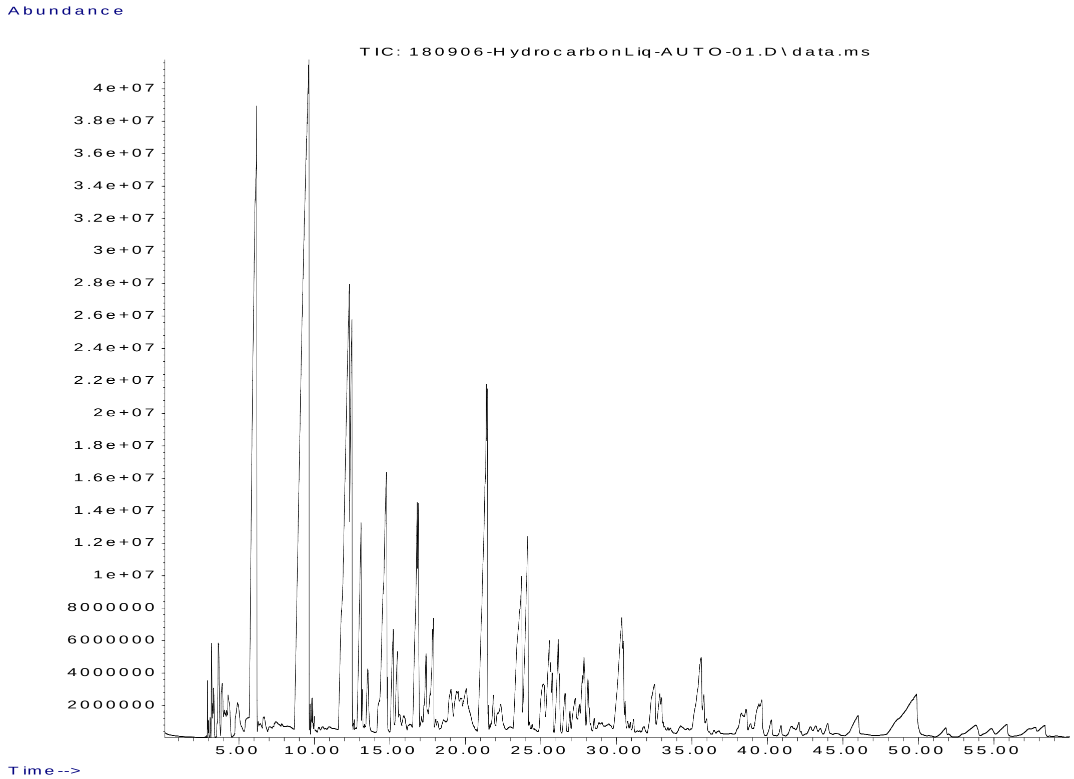

Supplementary Figure 3. MS chromatogram for liquid product collected on Ga/SiO_2 at 250°C and 30.6 atm.

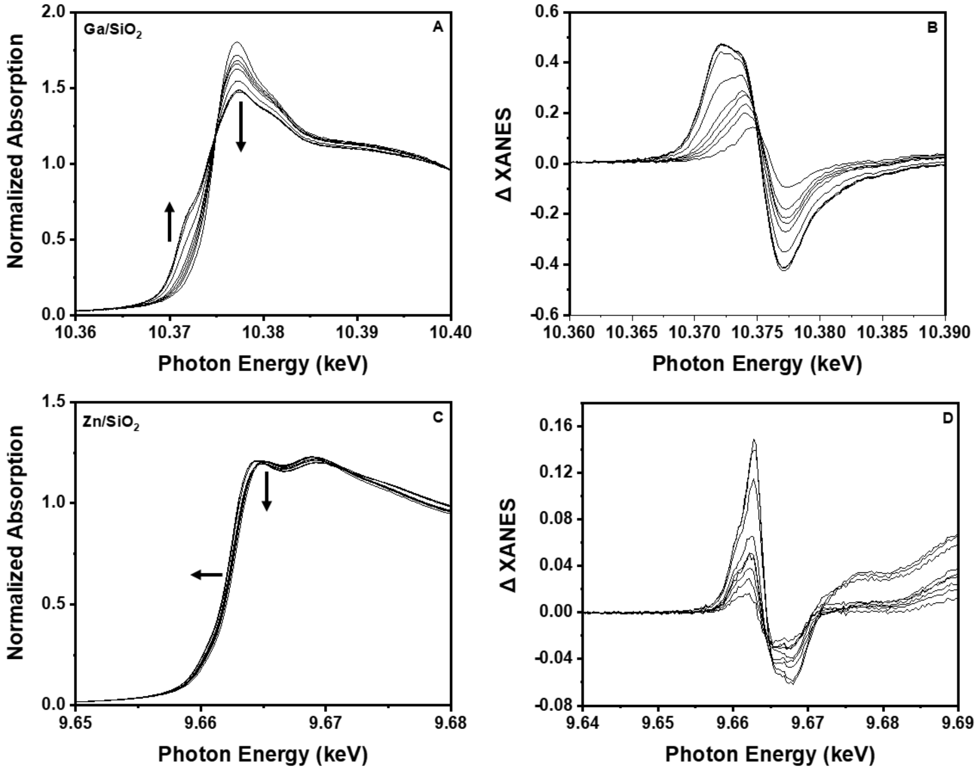

Supplementary Figure 4. ***In situ*** **XAS for Ga/SiO_2 and Zn/SiO_2 with increasing temperature to 550°C in pure H_2. a** Normalized Ga K edge XANES. **b** The difference between the high temperature scan and dehydrated structure for Ga/SiO_2. **c** The normalized Zn K edge XANES. **d** The difference between the high temperature scan and dehydrated structure for Zn/SiO_2.

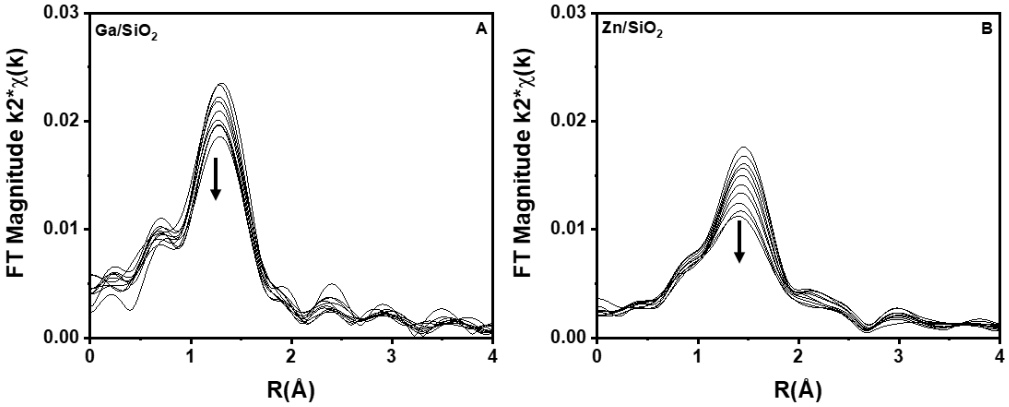

Supplementary Figure 5. k^2-weighted magnitude of the Fourier transform (Δk = 2.7-10.5 $Å^{-1}$) with increasing temperature to 550°C in pure H_2. a Ga/SiO_2. **b** Zn/SiO_2.

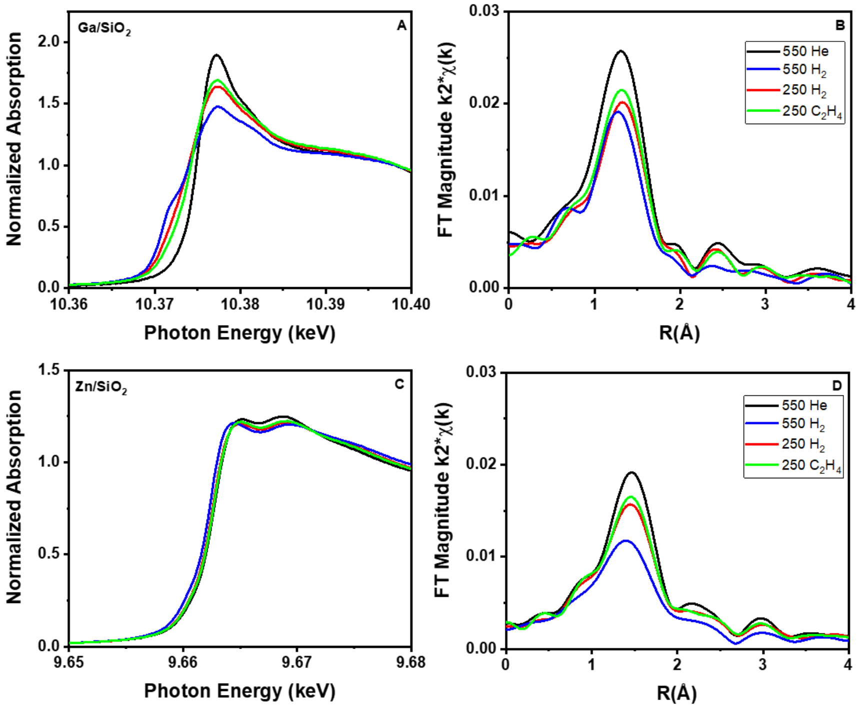

Supplementary Figure 6. The X-ray absorption spectra of catalysts pre-treated with different reacting gases. **a** XANES of Ga/SiO_2 in He 550°C (black), H_2 550°C (blue) and 250°C (red) and ethylene after H_2 at 250°C. **b** Fourier transform of the k^2-weighted EXAFS of Ga/SiO_2 in He 550°C (black), H_2 550°C (blue) and 250°C (red) and ethylene after H_2 at 250°C. **c** XANES of Zn/SiO_2 in He 550°C (black), H_2 550°C (blue) and 250°C (red) and ethylene after H_2 at 250C. **d** Fourier transform of the k^2-weighted EXAFS of Zn/SiO_2 in He 550°C (black), H_2 550°C (blue) and 250°C (red) and ethylene after H_2 at 250°C.

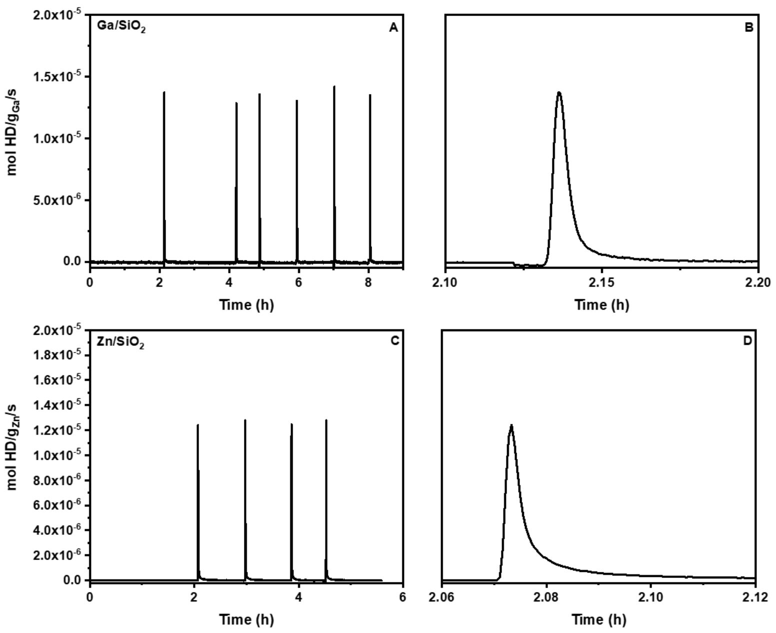

Supplementary Figure 7. Time on stream for gaseous HD formation at 250°C. **a** Total number of pulses for Ga/SiO_2. **b** Expanded view of the shape of a single pulse for Ga/SiO_2. Total number of pulses for Zn/SiO_2. **d** Expanded view of the shape of a single pulse for Zn/SiO_2.

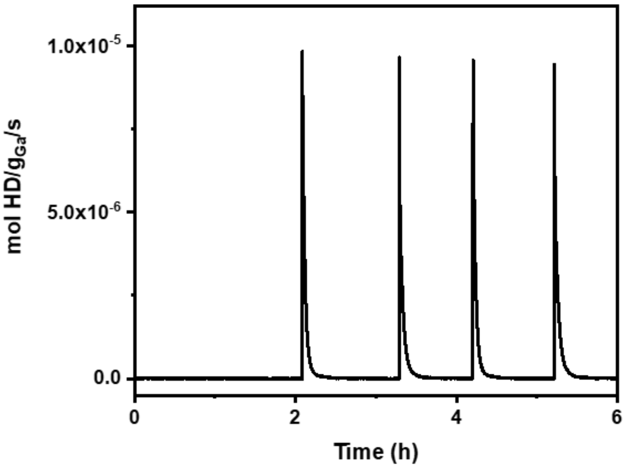

Supplementary Figure 8. Time on stream for gaseous HD formation on Ga/SiO_2 at 450°C.

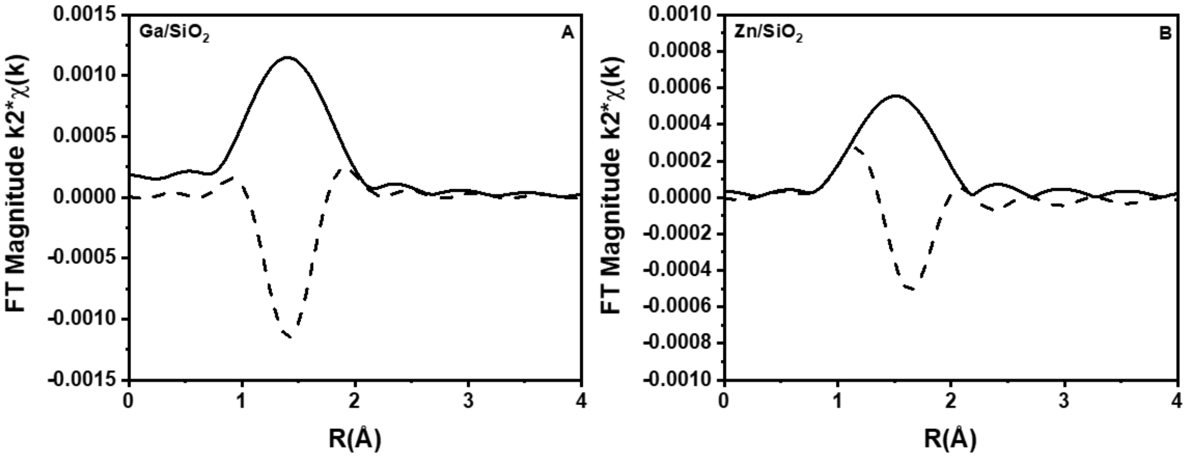

Supplementary Figure 9. Fourier transform of the k^2-weighted difference EXAFS after treatments in C_2H_4 minus in H_2 at 250°C. **a** Ga/SiO_2. **b** Zn/SiO_2.

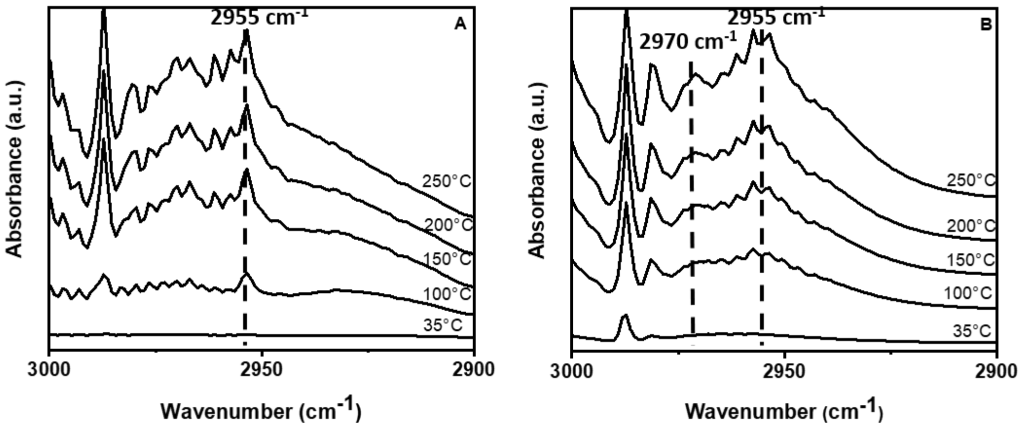

Supplementary Figure 10. Infrared spectroscopy C-H region of Ga/SiO_2 after exposure to pure C_2H_4 at increasing temperatures from 35°C to 250°C. a Pre-treated to 550°C in H_2. **b** dehydrated at 550°C Ga/SiO_2. Each spectrum is the ethylene catalyst at temperature minus the pre-treated (or dehydrated) catalyst spectrum (for example, Ga/$SiO_{2\ C2H4}$-Ga/$SiO_{2\ dehydrated}$).

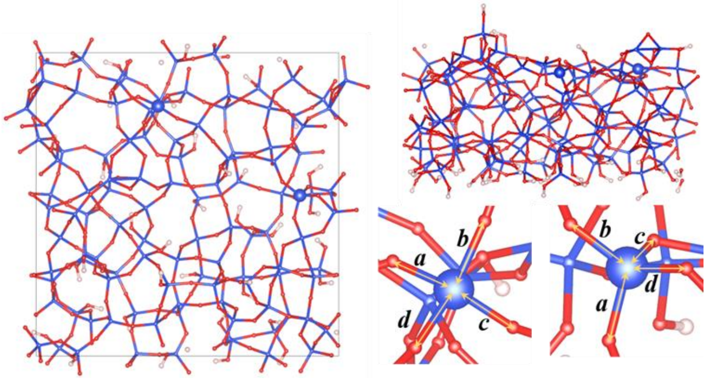

Supplementary Figure 11. Amorphous silica model with highlighted Si atoms to be substituted with Ga (Si = blue, O = red; H= white).

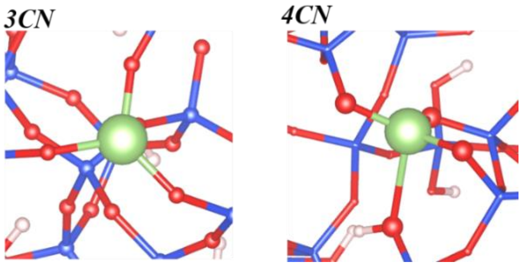

Supplementary Figure 12. Amorphous silica model with highlighted Si atoms to be substituted with Ga (Si = blue, O = red, H= white, Ga = green).

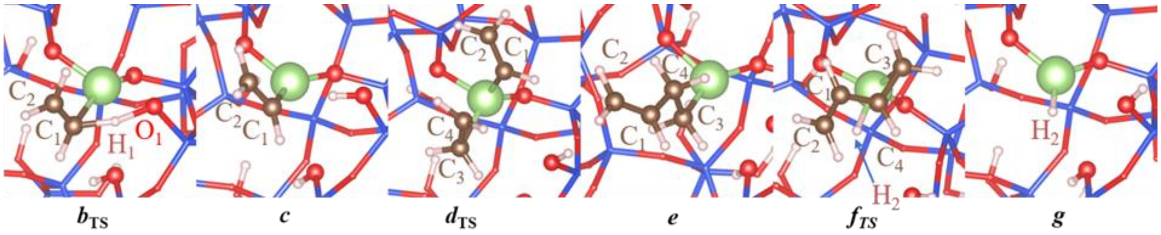

Supplementary Figure 13. Key intermediates and transition states for Ga-H formation at a 4CN Ga site (Si = blue, O = red, H= white, Ga = green).

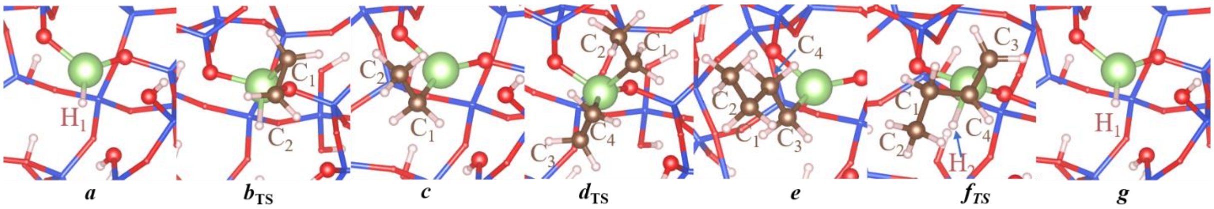

Supplementary Figure 14. Key intermediates and transition states of ethylene oligomerization on Ga-H generated from the 4CN Ga site (Si = blue, O = red, H= white, Ga = green).

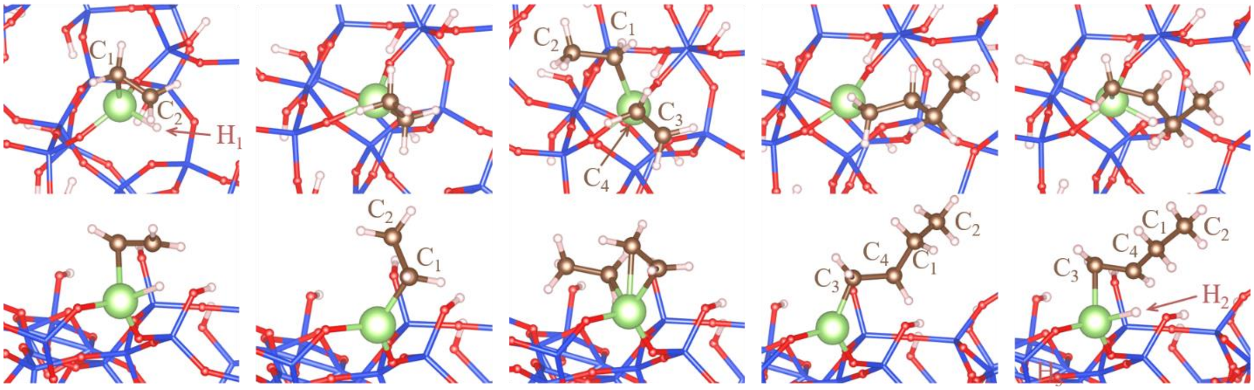

b_{TS} c d_{TS} e f_{TS}

Supplementary Figure 15. Top and side views of key intermediates and transition states of ethylene oligomerization on a Ga-H located in a 3CN environment (Si = blue, O = red, H= white, Ga = green).

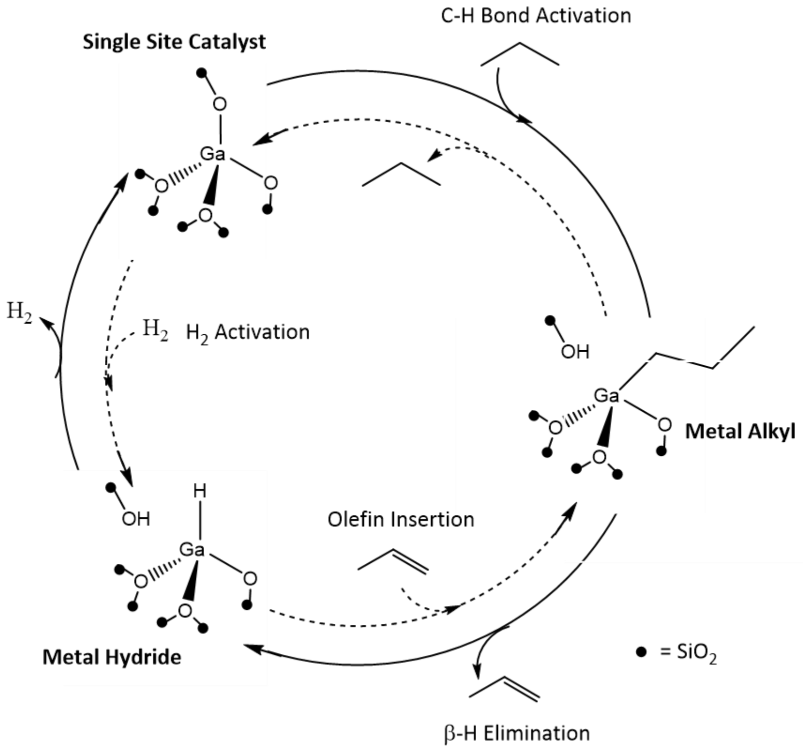

Supplementary Figure 16. Dehydrogenation (solid line) and hydrogenation (dashed line) reaction pathways.

Supplementary Tables

Supplementary Table 1. EXAFS fits of the as-prepared structures of Ni/SiO_2, Ga/SiO_2, and Zn/SiO_2 after dehydration at 550°C in He compared to bulk references at each edge.

Sample	Pre-edge Energy (keV)	XANES Energy (keV)	Scattering Path	CN[b]	R (Å)[c]	$\Delta\sigma^2$ ($Å^2$)	ΔE_o (eV)	
**NiO[a]**	8.3335	8.3410	Ni-O	6.0	2.09	-	-	Ni^{2+} (O_h)
**Ni/SiO_2**	8.3331	8.3421	Ni-O	4.0	2.03	0.006	-1.3	Ni^{2+} (Td)
**Ga_2O_3[a]**	-	10.3751	Ga-O	3.0	2.00	-	-	Ga^{3+} (T_d+O_h)
				2.0	1.83	-	-	
**$Ga(AcAc)_3$**	-	10.3793	Ga-O	6.0	1.93	-	-	Ga^{3+} (O_h)
**Ga/SiO_2**	-	10.3751	Ga-O	4.0	1.81	0.006	-2.2	Ga^{3+} (T_d)
**ZnO[a]**	-	9.6625	Zn-O	4.0	1.98	-	-	Zn^{2+} (T_d)
**Zn/SiO_2**	-	9.6628	Zn-O	4.0	1.95	0.006	-0.6	Zn^{2+} (T_d)

[a] NiO, Ga_2O_3, $Ga(AcAc)_3$, and ZnO are references of known structures

[b] The error in CN is ±10%
[c] The error in R is ±0.02 Å

Supplementary Table 2. Initial selectivity and TOR for Ga/SiO_2 and Zn/SiO_2 at 10% conversion during propane dehydrogenation performed at 550°C in 3% C_3H_8 and 2% H_2.

	Without H_2		***With H_2***	
	**Initial Selectivity (%)**	**TOR (1/h)**	**Initial Selectivity (%)**	**TOR (1/s)**
**Ga/SiO_2**	97	2.1	98	$4.4*10^{-3}$
**Zn/SiO_2**	99	0.9	99	$3.3*10^{-3}$

Supplementary Table 3. Initial TORs for Ga/SiO_2 and Zn/SiO_2 at 15% conversion during propylene hydrogenation performed at 200°C in 1% C_3H_6 and 3% H_2 after treatment various pretreatments.

**Pretreatment**	**Ga/SiO_2: Initial TOR (1/s)**	**Zn/SiO_2: Initial TOR (1/s)**
**200°C N_2**	$8.3*10^{-4}$	$2.2*10^{-3}$
**200°C H_2**	$6.9*10^{-3}$	$4.7*10^{-3}$
**550°C H_2**	$5.8*10^{-3}$	$6.7*10^{-3}$

Supplementary Table 4. Product selectivity at varying conversions for each catalyst during ethylene oligomerization at 250°C and 1 atm.

Catalyst	Conversion (%)[a]	Selectivity (%)				Rate (mol C_4H_8 molM^{-1} s^{-1})[b]	TOR (s^{-1})[c]
		Ethane (C_2H_6)	Butenes ($C_4^=$)	Hexenes ($C_6^=$)	Octenes ($C_8^=$)		
**Ni/SiO2**	1	1.0	89.2	9.7	0.5	8 x 10^{-4}	
	2	2.6	86.5	9.6	1.0	13 x 10^{-4}	
	3	1.4	86.1	11.8	0.4	15 x 10^{-4}	
	5	1.1	85.1	13.5	0.5	10 x 10^{-4}	
**Ga/SiO2**	1	4.5	87.0	2.6	5.8	3 x 10^{-4}	4 x 10^{-4}
	2	3.3	85.2	3.6	7.9	7 x 10^{-4}	9 x 10^{-4}
	3	1.8	75.9	16.8	6.4	7 x 10^{-4}	9 x 10^{-4}
**Zn/SiO2**	1	5.7	91.5	2.8	0.0	1 x 10^{-4}	5 x 10^{-4}
	2	11.6	87.3	1.1	0.0	1 x 10^{-4}	5 x 10^{-4}
	3	12.5	85.5	2.0	0.0	1 x 10^{-4}	5 x 10^{-4}

[a] Conversion was varied using different space velocities over 1g of catalyst in a 3/8 in diameter quartz reactor tube

[b] Rate was calculated by normalizing the mol C_4H_8/s produced by the total mol of metal on the catalyst ($rate = (mol\ C_4H_8)/(mol\ M * s)$)

[c] TOR was calculated by normalizing the rate by the percent of M-H formed, using the fraction of M-H obtained by H/D exchange experiments (described in Supplementary Figures 7, Supplementary Tables 8) ($TOR = (mol\ C_4H_8)/(mol\ M * \left(\frac{\%M-H}{100}\right) * s)$)

Supplementary Table 5. Product selectivity and conversion for ethylene oligomerization at 250°C and 30.6 atm.

Catalyst	Conversion (%)	Selectivity (%)					Rate (mol C_4H_8 molM^{-1} s^{-1})	TOR (s^{-1})
		Ethane (C_2H_6)	Butenes ($C_4^=$)	Hexenes ($C_6^=$)	Octenes ($C_8^=$)	C_{10}+		
**Ni/SiO2**	20.7	0.6	86.2	11.1	2.9	0.0	7 x 10^{-2}	
**Ga/SiO2**	20.6	0.6	74.2	16.1	4.9	4.2	8 x 10^{-2}	1 x 10^{-1}
**Zn/SiO2**	15.2	0.5	96.0	0.8	0.0	0.0	5 x 10^{-2}	3 x 10^{-1}

Supplementary Table 6. Product selectivity at varying conversions for each catalyst during propylene oligomerization at 250°C and 1 atm.

Catalyst	Conversion (%)[a]	Selectivity (%)							Rate (mol C_6H_{12} $molM^{-1}$ s^{-1})[b]	TOR (s^{-1})[c]
		Methane (CH_4)	Ethylene ($C_2^=$)	Propane (C_3H_8)	Butenes ($C_4^=$)	Hexenes ($C_6^=$)	Nonenes ($C_9^=$)	C_{12}^+		
Ga/SiO_2	1	0.1	5.5	3.9	6.3	8.9	75.1	0.2	9 x 10^{-4}	1 x 10^{-3}
	3	0.1	5.9	6.3	6.7	7.8	71.9	1.3	12 x 10^{-4}	2 x 10^{-3}
	5	0.2	7.4	5.3	7.4	16.1	52.3	11.3	9 x 10^{-4}	1 x 10^{-3}
	8	0.2	10.4	7.7	8.9	23.5	43.4	5.9	14 x 10^{-4}	2 x 10^{-3}
Zn/SiO_2	1	0	3.8	9.2	3.2	50.5	28.1	5.2	8 x 10^{-4}	1 x 10^{-3}
	3	0	2	6.1	1.3	52.2	29.6	8.8	7 x 10^{-4}	1 x 10^{-3}
	5	0	5.9	8.9	5.4	36.7	30.8	12.3	4 x 10^{-4}	1 x 10^{-3}
	8	0	6.2	6.2	6.6	41.4	30	9.6	4 x 10^{-4}	1 x 10^{-3}

[a]Conversion was varied using different space velocities over 1g of catalyst in a 3/8 in diameter quartz reactor tube

[b] Rate was calculated by normalizing the mol C_6H_{12}/s produced by the total mol of metal on the catalyst ($rate = (mol\ C_6H_{12})/(mol\ M * s)$)

[c] TOR was calculated by normalizing the rate by the percent of M-H formed, using the fraction of M-H obtained by H/D exchange experiments (described in supplementary Figures 7, Supplementary Table 8) ($TOR = (mol\ C_6H_{12})/(mol\ M * \left(\frac{\%M-H}{100}\right) * s)$)

Supplementary Table 7. Ga K and Zn K edge XAS fitting parameters for the metal-oxygen bonds in Ga/SiO_2 and Zn/SiO_2 after treatment in pure H_2 at successively increasing temperatures.

Sample	Pretreatment Conditions	XANES Energy (keV)	Scattering Path	CN	R (Å)	$\Delta\sigma^2$ ($Å^2$)	ΔE_o (eV)
Ga/SiO_2	[a] He 550°C	10.3751	Ga-O	4.0	1.81	0.006	-2.2
	H_2 250°C	10.3750	Ga-O	3.5	1.81	0.006	-1.0
	H_2 550°C	10.3747	Ga-O	3.2	1.80	0.004	-1.9
	C_2H_4 250°C	10.3750	Ga-O	3.5	1.81	0.006	-2.2
Zn/SiO_2	[a] He 550°C	9.6628	Zn-O	4.0	1.95	0.006	-0.6
	H_2 250°C	9.6627	Zn-O	3.6	1.93	0.006	-0.9
	H_2 550°C	9.6625	Zn-O	3.2	1.93	0.004	-1.7
	C_2H_4 250°C	9.6627	Zn-O	3.4	1.93	0.006	-1.0

[a] Fits for the dehydrated catalysts were copied from Supplementary Table 1 and are used for comparison to the fits after treatment

Supplementary Table 8. HD moles quantified by H_2/D_2 exchanges at 250°C and corrected to remove non-metal contributions.

**Sample**	**Moles HD evolved in consecutive H_2 or D_2 step changes / mol/ g**	
	**$H_2 \rightarrow D_2$**	**$D_2 \rightarrow H_2$**
**Ga/SiO_2**	0.74	0.73
	0.64	0.71
	0.64	0.76
**Zn/SiO_2**	0.21	0.22
	0.16	0.18

Supplementary Table 9. HD moles quantified by H_2/D_2 exchanges at 450°C and corrected to remove non-metal contributions.

**Sample**	**Moles HD evolved in consecutive H_2 or D_2 step changes / mol/ g**	
	**$H_2 \rightarrow D_2$**	**$D_2 \rightarrow H_2$**
**Ga/SiO_2**	1.12	1.10
	1.04	1.07

Supplementary Table 10. Difference EXAFS (C_2H_4 - H_2) fitting parameters at the Ga K and Zn K edges to identify the number of metal-carbon bonds at 250°C.

**Sample**	**Scattering Path**	**CN**	**R (Å)**	**$\Delta\sigma^2$ (Å^2)**	**ΔE_o (eV)**
**Ga/SiO_2**	Ga-C	0.4	1.94	0.005	6.1
**Zn/SiO_2**	Zn-C	0.2	2.01	0.005	3.2

Supplementary Table 11. Bond distances and free energies of key intermediates and transition states during Ga-H formation at a 4CN Ga site. The calculation of adsorption energy used the reference of the empty Ga site and the gaseous ethylene molecule at 1 atm and 250°C. See below for the entropy calculation procedure. TS indicates a transition state.

**Intermediate***	**Bond**	**Length (Å)**	**Free Energy (eV)**	**Energy (eV)**	**Entropy (J mol^{-1} K^{-1})**
a Empty Ga site	-	-	0	0	66.0
$\boldsymbol{b}_{TS}$	Ga – C_1	2.15	1.69	1.26	240.38 (product-like geometry)
	O_1 – H_1	1.33			
c	Ga – C_1	1.96	0.36	-0.06	240.38
	O_1 – H_1	0.97			
$\boldsymbol{d}_{TS}$	Ga – C_1	2.11	1.83	0.66	330.32 (product-like geometry)
	Ga – C_3	2.06			
	Ga – C_4	2.35			
e	Ga – C_3	1.98	-0.21	-1.36	330.32
$\boldsymbol{f}_{TS}$	Ga – C_3	2.04	1.92	0.70	330.32 (reactant-like geometry)
	Ga – H_2	1.73			
g (Ga-H)	Ga – H_2	1.55	0.15	-0.50	136.77

* Intermediates shown in Supplementary Figure 13

Supplementary Table 12. Bond distances and free energies of key intermediates during ethylene oligomerization on Ga-H generated from 4CN Ga. The calculation of adsorption energy used the reference of the empty Ga-H site and the gaseous ethylene molecule at 1 atm and 250°C. See below for the entropy calculation procedure. TS indicates a transition state.

Intermediate*	Bond	Length (Å)	Free Energy (eV)	Energy (eV)	Entropy (J mol^{-1} K^{-1})
a (Ga-H)	Ga – H_1	1.55	0	0	70.7
$\boldsymbol{b}_{TS}$	Ga – C_1	2.08	1.82	1.13	179.9 (product-like geometry)
	Ga – H_1	1.72			
c	Ga – C_1	1.98	-0.52	-1.20	179.9
$\boldsymbol{d}_{TS}$	Ga – C_1	2.23	1.54	0.19	283.8 (product-like geometry)
	Ga – C_3	2.04			
	Ga – C_4	2.24			
e	Ga – C_3	1.98	-0.79	-2.14	283.8
$\boldsymbol{f}_{TS}$	Ga – C_3	2.03	1.24	-0.06	283.8 (reactant-like geometry)
	Ga – H_2	1.75			
g (Ga-H)	Ga – H_1	1.55	-0.11	-1.08	70.7

* Intermediates shown in Supplementary Figure 14.

Supplementary Table 13. Bond distances and free energies of key intermediates during ethylene oligomerization on Ga-H generated from 3CN Ga. The calculation of adsorption energy used the reference of the empty Ga-H site and the gaseous ethylene molecule at 1 atm and 250°C. See below for the entropy calculation procedure. TS indicates a transition state.

**Intermediate***	**Bond**	**Length (Å)**	**Free Energy (eV)**	**Energy (eV)**	**Entropy (J mol^{-1} K^{-1})**
Ga-H	Ga – H_1	1.55	0	0	49.9
$\boldsymbol{b}_{TS}$	Ga – C_1	2.10	1.49	0.87	179.3 (product-like geometry)
	Ga – H_1	1.71			
c	Ga – C_1	1.98	-0.75	-1.36	179.3
$\boldsymbol{d}_{TS}$	Ga – C_1	2.28	1.12	-0.02	303.5 (product-like geometry)
	Ga – C_3	2.04			
	Ga – C_4	2.26			
e	Ga – C_3	1.98	-1.25	-2.39	303.5
$\boldsymbol{f}_{TS}$	Ga – C_3	2.09	1.11	-0.03	303.5 (reactant-like geometry)
	Ga – H_2	1.70			
g (Ga-H)	Ga – H_1	1.55	-0.11	-1.08	49.9

* Intermediates shown in Supplementary Figure 15

Supplementary Table 14. Examples of entropy contributions from the vibrations with wavenumbers less than 150 cm^{-1} (T = 250 °C).

Ga-ethyl (c in Supplementary Fig. 14)			Ga-n-butyl (e in Supplementary Fig. 14)		
**Wavenumber, cm^{-1}**	**Description**	**Entropy contribution, $J\ mol^{-1}\ K^{-1}$**	**Wavenumber, cm^{-1}**	**Description**	**Entropy contribution, $J\ mol^{-1}\ K^{-1}$**
58.3	Translation	35.4	24.9 (imaginary)	Translation	38.2
83.2	Ga vibration	-	24.7	Translation	38.2
101.1	Translation	35.4	56.4	Ga vibration	-
125.4	Ga vibration	-	86.9	Rotation	34.2
156.1	Rotation	25.6	104.6	Rotation	34.2
			131.6	Ga vibration	-
			157.7	Translation (adsorbate + Ga)	41.5

Supplementary Methods

Elemental Analysis. Atomic absorption spectroscopic analysis of the Ni, Ga, and Zn loading was carried out on a Perkin-Elmer Analyst 300 Flame Atomic Absorption Spectrometer using air-acetylene flame. The samples were firstly dissolved in 40% HF aqueous solution and diluted to 1-2 ppm. The analysis was done using the 232.0 nm line. The Ni loading in Ni/SiO_2 was 2.7%. The Ga loading in Ga/SiO_2 was 4.0%. The Zn loading in Zn/SiO_2 was 4.0%.

Catalyst Testing. Propane Dehydrogenation and Propylene Hydrogenation: Catalyst performance tests for hydrogenation and dehydrogenation were performed in a fixed bed reactor with a quartz reactor tube of 3/8-inch OD. Dehydrogenation was performed in 3% C_3H_8 and 2% H_2 balanced in N_2 at 550°C on 1 g of catalyst using varying total flow rates of the same gas composition ranging from 41 ccm to 165 ccm to vary conversion (GHSV = 0.32 s^{-1} – 1.28 s^{-1}). Before each dehydrogenation test, the catalyst was pretreated in flowing N_2 while the temperature ramped to 550°C. The temperature was stabilized for 1 hour prior to starting the reaction.
Hydrogenation was performed in 1% C_3H_6, 3% H_2, balanced with N_2 at 200°C on 250 mg of catalyst diluted to 1 g with silica using a total flow rate of 104 ccm (GHSV = 0.81 s^{-1}). Before each hydrogenation test, the catalysts were pretreated using one of the following pretreatments: 1) ramped to 200°C in flowing N_2, 2) ramped to 200°C in flowing H_2, or 3) ramped to 550°C in flowing H_2 and then cooled to 200°C for the reaction. In each case, the temperature was stabilized for 1 hour prior to starting the reaction.
Dehydrogenation and hydrogenation products were analyzed with a Hewlett Packard (HP) 6890 Series gas chromatograph (GC) using a flame ionization detector (FID) with a Restek Rt-Alumina Bond/Na2SO4 GC column (30 m in length, 0.32 mm ID, and 0.5 µm film thickness).

X-Ray Absorption Spectroscopy. *In-situ* X-ray Absorption Spectroscopy (XAS) experiments were performed at the 10-BM sector at the Advanced Photon Source at Argonne National Laboratory. All measurements were performed at the Zn K (9.659 keV) or Ga K (10.3670 keV) edge in transmission mode in fast scan from 250 eV below the edge to 550 eV above the edge. Ga2O3 (10.3751 keV) and Zn foil was used to calibrate the x-ray energies at the Ga and Zn K edges, respectively. Samples were pressed into a stainless-steel sample holder and placed in a sample cell. The cell was sealed and the sample was treated successively by 500°C in He (dehydrated) or in in pure H_2 or ethylene from room temperature to 550°C.
The absorption data was processed using the WinXAS v.3.2 software to find the coordination number and bond distance using standard procedures. The phase and amplitude functions for Ga-O and Zn-O were extracted from theoretical Feff6 calculations using Ga(Acetylacetonate)$_3$ (CN=6, R=1.96 Å) and ZnO (CN=4, R=1.98 Å) as references. A least squared fit the first shell of r- space and isolated q- space were performed on the k^2 weighted Fourier transform data over the range 2.7 to 10 $Å^{-1}$ in each spectrum to fit the magnitude and imaginary components.

Density Functional Theory. Periodic amorphous silica model is used to analyze the energetics of ethylene oligomerization. The amorphous model was adapted using models described in literature.[1] To model the four-coordinated Ga sites, Si was substituted with Ga, and an additional proton was added to a nearby oxygen atom to maintain charge balance (all possible sites for adding

the proton were considered, and the site with lowest energy was chosen for further analysis). All DFT calculations are performed with self-consistent and periodic density functional theory using Vienna Ab-initio Simulation Package (VASP). The BEEF-vdw exchange-correlation functional using projector augmented wave (PAW) pseudopotentials was used. A dipole correction was applied parallel to the plane of the slab to reduce image−image interaction errors. A cutoff energy of 400 eV was considered with a force-convergence criterion of 20 meV $Å^{-1}$.

Supplementary Notes

Supplementary Note 1

Catalyst Preparation and Initial Structure. Silica supported Ga and Zn single site catalysts were prepared using previously published synthesis methods.[2,3] Ga/SiO_2 was prepared by impregnation using a citric acid chelating agent. Zn/SiO_2 was prepared using strong electrostatic absorption (SEA). All solutions were pH adjusted to 11. Due to the slightly acidic nature hydroxyl groups on groups on SiO_2, at high pH deprotonation of the SiOH groups leads to a negative surface charge. This allows a strong interaction between the cationic ligands and the negative surface, resulting in homogeneously dispersed metal ions on the SiO_2 surface. Ni/SiO_2 was prepared using a similar procedure to that of Zn/SiO_2. Atomic absorption spectroscopy revealed that the metal content was 2.6 wt%, 4.1 wt%, and 3.1 wt% metal for Ga/SiO_2, Zn/SiO_2, and Ni/SiO_2 respectively.

The pre-reaction catalyst structure was determined by *in situ* X-ray absorption spectroscopy (XAS), including both XANES and EXAFS on Ga/SiO_2 and Zn/SiO_2 and compared to that of Ni/SiO_2. The catalysts were dehydrated at 550°C in He and compared to known reference compounds at each metal edge (Supplementary Figure 1). The EXAFS fits of each catalyst are given in Table 1. The XANES was used to determine the oxidation state of each catalyst, while the EXAFS was used to identify the coordination environments prior to any catalytic treatment. On Ni/SiO_2, the Ni K-edge XANES pre-edge energy (8.3331 keV) is slightly lower than that of NiO (8.3335 keV) (Supplementary Figure 1a). The NiO XANES is consistent with Ni^{2+} in an octahedral coordination, while the pre-edge energy of the former is consistent with Ni^{2+} in s tetrahedral geometry.[4] The first shell EXAFS fit of Ni/SiO_2 has 4 Ni-O bonds at 2.03 Å, while NiO has Ni-O bonds at 2.09 Å (Supplementary Figure 1b). The EXAFS Ni/SiO_2 and is consistent with the single site Ni^{2+} previously reported.[4–6] Since Ga/SiO_2 and Zn/SiO_2 have d^{10}-electron configuration, there is no pre-edge feature in the XANES region. The XANES energy relative to known references was used to identify the oxidation state of each catalyst. The XANES energy of Ga^{3+} is also dependent on the coordination geometry[7,8]. Tetrahedral (T_d) Ga^{3+} (10.3745 keV) has a XANES energy, lower than that in octahedral (O_h) coordination (10.3793 keV). Ga_2O_3 has 50% T_d and 50% O_h Ga^{3+}.[9] Since the edge energy of T_d is lower than O_h Ga^{3+}, the XANES of Ga_2O_3 is 10.3751 keV, (Supplementary Figure 1c). The XANES energy of $Ga(AcAc)_3$, Oh Ga^{3+} is 10.3765 keV. The XANES energy (10.3751 keV) of Ga/SiO_2 is consistent with Td Ga^{3+}. The Ga coordination geometry also affects the Ga-O bond distance. The bond distance of Ga-O in a Td Ga compound is about 1.83 Å; while in Oh compounds Ga-O bonds are longer, about 1.90-2.00 Å.[9] The k^2-weighted magnitude of the EXAFS of Ga/SiO_2 is lower than that of $Ga(AcAc)_3$ (6 Ga-O at 1.93 Å) (Supplementary Figure 1d). The fit of Ga/SiO_2 EXAFS, Supplementary Table 1, indicates about 4 Ga-O bonds at 1.81 Å, both consistent with Td Ga^{3+}. The absence of Ga-O-Ga bonds also suggests isolated Ga^{3+} bound to the support. Similarly, the XANES energy for Zn/SiO_2

(9.6628 keV) is similar to that of ZnO (9.6625 keV) (Supplementary Figure 1e) consistent with Zn^{2+} oxidation state. The magnitude of the k^2-weighted EXAFS of Zn/SiO_2 has 4 Zn-O bonds at 1.96 Å and is also similar to ZnO (4 Zn-O at 1.98 Å). The absence of Zn-O-Zn higher shell coordination in the catalyst, however, suggests a single site Zn^{2+} structure.

Supplementary Note 2

Propane Dehydrogenation and Propylene Hydrogenation. The turnover rate and selectivity for C_3H_8 dehydrogenation was determined with and without H_2 cofeed. Dehydrogenation in the presence of H_2 is a more rigorous way to test for selective performance towards the production of C_3H_6. Both Ga/SiO_2 and Zn/SiO_2 were highly selective towards olefins (> 95%) up to 20% conversion, even in the presence of H_2. High C_3H_6 selectivity was maintained as a function of conversion. In addition, the catalysts were stable under reaction conditions at 550°C for over 6 hours. The turnover rates (TOR) for each catalyst were calculated by normalizing the amount of C_3H_6 production by the moles of metal on the surface of the catalyst (2.6% for Ga/SiO_2 and 4.0% for Zn/SiO_2). The TOR is shown for 10% conversion (Supplementary Table 2). The TOR on Ga/SiO_2 and Zn/SiO_2 are of the same order of magnitude, and co-feeding H_2 does not significantly change the TOR during dehydrogenation. These results are consistent with what has previously been reported.[3,8] Both catalysts also perform the microscopic reverse reaction of propane dehydrogenation (propylene hydrogenation) at lower reaction temperatures. Propane was the only product indicating few side reactions occurred at 200°C. The influence of H_2 pretreatment on C_3H_6 hydrogenation activity at 200°C was explored to demonstrate how the catalysts behave in the presence of H_2 (Supplementary Table 3). The initial TOR of the catalysts treated in H_2 at 200°C and 550°C was compared to that of the dehydrated catalyst (200°C N_2). Pretreatment in H_2 leads to higher initial TOR than what was achieved on the dehydrated catalyst. However, after approximately 30 minutes, the TOR for all pretreatment conditions stabilized to approximately the same value.

Supplementary Note 3

Olefin Oligomerization. Like the structural characterizations, the catalytic rates and selectivities for propane dehydrogenation and propylene hydrogenation of these Ga/SiO_2 and Zn/SiO_2 catalysts are very similar to reported performance, reaction intermediates and elementary reaction steps suggesting that these may also be catalytic for olefin oligomerization. The catalytic performance in both ethylene and propylene oligomerization was investigated at 250°C. Under these reaction conditions, SiO_2 does not facilitate catalytic transformations.

Ethylene Oligomerization. Ethylene oligomerization was performed at 250°C and 1 atm, where low conversions were achieved. The conversion was varied using different space velocities over 1 g of catalyst. The catalyst stability as a function of time on stream is shown in Supplementary Figure 2. At 250°C, both Ga/SiO_2 and Zn/SiO_2 were shown to be stable for at least 25 hours. The stability at a higher reaction temperature of 450°C was also evaluated and the catalysts were shown to not deactivate within 40 hours. However, because Ni/SiO_2 is not structurally stable at 450°C, in that Ni^{2+} reduces to Ni^0 and becomes inactive, all further catalyst testing was performed at 250°C. Although Ni/SiO_2 had the potential to reach higher conversions than Ga/SiO_2 and Zn/SiO_2, the conversion was limited to 5% so that direct comparisons in product distributions could be made (Supplementary Table 4).

In Supplementary Tables 4-6, two rates are reported. First, the rate of butene (or dimer) formation, in mol/s, was normalized by the total number of moles M on the catalyst. For Ni/SiO_2 this was 2.7 wt% and for Ga/SiO_2 and Zn/SiO_2, it was 2.6 and 4.0 wt%, respectively. The TOR was reported by normalizing the rate by the amount of M-H formed, as obtained by H/D exchange experiments, described in Supplementary Figures 7 and Supplementary Tables 8. This value was not obtained for Ni/SiO_2 but was determined to be 70% and 19% for Ga/SiO_2 and Zn/SiO_2, respectively. During oligomerization, the M-H is thought to be the active intermediate. This shows that the rate of Ga/SiO_2 and Ni/SiO_2 are about three times higher than Zn/SiO_2. However, when the rate is normalized by the fraction of M-H formed, the rates are similar.

Like Ni/SiO_2, Zn/SiO_2 favored the formation of butene from ethylene. However, the rate of the latter ($1*10^{-4}$ s^{-1}) was lower than the former ($10*10^{-4}$ s^{-1}). Ga/SiO_2 ($7*10^{-4}$ s^{-1}), on the other hand, had a rate more comparable to that of Ni/SiO_2 and facilitated the formation of higher molecular weight hydrocarbons. To increase the reaction rate, and therefore drive the conversion to a higher value, a higher concentration of ethylene was used by increasing the total reaction pressure to 30.6 atm. Through this, higher molecular weight products in the liquid range were favored. Here, the reaction rates were improved by two orders of magnitude such that those on all three catalysts were similar (and to the same order of magnitude). Although Ni/SiO_2 formed predominantly butenes (86%), the higher ethylene concentration promoted the formation of some hexenes (11%) and octenes (3%). Zn/SiO_2 almost exclusively produced butenes (96%) with only some hexenes (0.8%) and no octenes. Ga/SiO_2 formed a larger amount of higher molecular weight hydrocarbons than Ni/SiO_2 including 74% butenes, 16% hexenes, 5% octenes, and 4% higher than C10.

Although Supplementary Table 5 quantifies only the gas phase products, the liquid products were accumulated during the reaction and analyzed offline by GC-MS after the reaction to develop a qualitative understanding of the hydrocarbon species being formed. The liquid products collected cannot be correlated to a specific time on stream, though the final liquid product showed evidence of at least a small concentration of higher molecular weight products. Liquid products were diluted with THF and separated using a standard DB-1 GC column. Supplementary Figure 3 shows the total chromatogram collected using mass spectrometry. The GC-MS shows the formation of linear hydrocarbons up to at least C15 and the formation of trace amounts of aromatics. Additional products with a higher molecular weight did not elude from the column and required overnight baking. The specific identify of these molecules could not be determined.

Propylene Oligomerization. To further investigate the ability of these single site catalysts to activate C-H bonds, propylene oligomerization was performed at 250°C and 1 atm on Ga/SiO_2 and Zn/SiO_2. Though, the C-H bond activation energy of propylene is lower than that of ethylene, which could lead to higher reaction rates. Through this, the formation of higher molecular weight hydrocarbons is expected (Supplementary Table 6). Higher conversion of propylene was observed than ethylene on both Ga/SiO_2 and Zn/SiO_2. In addition, small amounts of products from side reactions were also observed. On Ga/SiO_2, there is a high selectivity towards $C_6^{=}$ (60-80%) and moderate selectivity towards $C_9^{=}$ (5-15%) and higher carbon numbers (1-5%). Zn/SiO_2 had a rate approximately 3 times lower than Ga/SiO_2, but maintained a similar product distribution, however, no products with carbon numbers greater than 9 were observed.

Supplementary Note 4

Determination of the Schultz Flory Coefficient. A Schulz Flory coefficient inherently compares the rate of olefin insertion, or propagation (α), and β-hydride elimination, or termination (1-α). α is commonly used to predict the formation of higher molecular weight hydrocarbons on oligomerization catalysts. This means that catalysts with low α favor the formation of lower molecular weight hydrocarbons, and the rate of termination is higher than the rate of propagation. Those with higher α favor the formation of higher molecular weight hydrocarbons, and the rate of propagation is higher than the rate of termination.

The Schultz Flory coefficient (α) was calculated for Ni/SiO_2, Ga/SiO_2, and Zn/SiO_2 during ethylene and propylene oligomerization using the e^{slope} of the Schultz Flory distribution.[10] For ethylene oligomerization, it was found that $\alpha_{Ni} \sim \alpha_{Zn} < \alpha_{Ga}$. This is consistent with the product distributions listed in Supplementary Table 2 and implies that the β-H elimination elementary step on Ni/SiO_2 and Zn/SiO_2 is fast relative to olefin insertion, thus both selectivity favors lower molecular weight products, *e.g.* $C_4^{=}$. For Ga/SiO_2, the opposite is true. For propylene oligomerization, it was also found that $\alpha_{Zn} < \alpha_{Ga}$ though both values were higher than those on the former. This implies that there is a greater likelihood of making higher molecular weight products with propylene than ethylene and could be due to the lower C-H bond activation energy.[11]

Supplementary Note 5

Discussion of Oligomerization Products. Oligomerization on Ga^{3+} and Zn^{2+} single site catalysts were compared to that on Ni^{2+} all supported on SiO_2 (non-acidic). Silica supported catalysts are not expected to result in products containing odd carbon numbers because cracking ($C_8^{=} \rightarrow iC_4^{=}$ or $C_3^{=} + iC_5^{=}$) is not expected in the absence of H^+ sites. Consistent with the lack of acidity of the silica support, no isobutene is produced during ethylene oligomerization and higher molecular weight products were not formed during propane dehydrogenation. Each of these catalysts formed varying product distributions of linear olefins including an equilibrium distribution of $C_4^{=}$ (1-butene, cis-2-butene, and trans-2-butene), $C_6^{=}$ and $C_8^{=}$ isomers, in addition to trace amounts of interesting products like methane and ethane. The formation of paraffins could be explained by the formation of butadiene during initiation.

Supplementary Note 6

X-ray Absorption Spectroscopy (XAS). *In situ* XAS on both Ga/SiO_2, and Zn/SiO_2 was performed to capture the conditions at which relevant reaction intermediates (M-H and M-R) were formed. In H_2 with increasing temperatures from room temperature to 550°C, there are continual changes in the shape of the Ga and Zn K-edge XANES. For Ga/SiO_2 (Supplementary Figure 4a), the white line intensity decreases and there is a formation of a feature before the edge. Similar, though less obvious, changes were observed on Zn/SiO_2 (Supplementary Figure 4c). Changes in XANES were isolated by subtraction of the dehydrated sample from the sample treated in H_2 (Supplementary Figures 4b, 4d) and are due to changes in electron density on the active metal center.[12] This has also previously been assigned to formation of Ga^{3+} hydride intermediates.[2] Zn^{2+} hydrides have also been proposed based on DFT modeling.[3] For both catalysts, at 250°C, the changes are small but increase with increasing temperature in H_2. This matches the trends observed in the Fourier transform magnitude (FT mag). With increasing temperature, the intensity of the FT mag decreases, corresponding to a loss of M-O bonds (Supplementary Figures 5a, 5b).

This was fit in Supplementary Table 7 to show a decrease from 4.0 to 3.2 Ga-O bonds between room temperature to 550°C respectively. There was also a loss of 4.0 to 3.2 Zn-O bonds between room temperature to 550°C respectively.

When the temperature was decreased to 250°C from 550°C in pure H_2, the changes to the XANES were reversed. This was previously interpreted as a temperature dependent equilibrium between the initial single site structure containing 4 M-O bonds and a metal hydride intermediate containing 3 M-O and 1 M-H bonds.[8,12] The XANES spectra incrementally shifted back to the initial position while the intensity of the Fourier transform magnitude (EXAFS) continued to increase with decreasing temperature, suggesting an increasing M-O coordination number. When the temperature was stabilized at 250°C in H_2, and the structure stopped changing, the treatment gas composition was switched from pure H_2 to pure C_2H_4, which led to restoration of the XANES spectra back to its original position (Supplementary Figures 6a and 6c). This suggests that the metal hydride can facilitate olefin insertion thus forming small concentrations of metal alkyls. Likewise, there was a slight increase in the FT mag, suggesting that a new M-C bond was formed (Supplementary Figures 6b, 6d). Due to the similarly between C and O, M-O and M-C bonds cannot be distinguished by XAS. However, a fit of the FT mag showed that the Ga-O coordination number increased from 3.2 to 3.5 and Zn-O increased from 3.2 to 3.6 after exposure to ethylene (Supplementary Table 5). However, the XANES and EXAFS spectra are not completely restored to their original position because under the catalytic cycle, there is likely a combination of metal hydride and metal alkyl intermediates which have competing effects on the resulting spectra. These changes were isolated, and a difference analysis was performed to verify the number of new M-C bonds (Supplementary Figure 9, Supplementary Table 10).

An attempt to deconvolute the M-O and M-C bonds was made through difference analysis. To identify the number of metal carbon bonds, a difference between the C_2H_4 and H_2 treated catalysts at 250°C was performed. Although the resulting feature is small (Supplementary Figure 9a, 9b)), it can be fit (Supplementary Table 10) to reveal 0.4 and 0.2 metal-carbon bonds in Ga/SiO_2 and Zn/SiO_2 respectively.

Isotopic Exchange. H_2/D_2 isotopic exchange experiments were used to count the number of metal sites that formed a metal hydride. Each sample was dehydrated at 550°C in He. The sample was cooled to 250°C in He before being exposed to 5% H_2/Ar, thus forming some M-H bonds. After 1 hour, the H_2 feed was replaced with 5% D_2/Ar to form M-D bonds and gaseous HD. After 1 hour, the D_2 feed was replaced by H_2 to form M-H bonds and gaseous HD. This was performed to quantify the number of species that reversibly exchange isotopes. Supplementary Figure 7 shows the time on stream for gaseous HD formation after sequential $H_2 – D_2 – H_2$ switches. The measured HD molar flow was baseline corrected and normalized by the mass of metal (Ga or Zn) used for the experiment. The area under the curve corresponds to the number of metal sites which reversibly undergo H/D exchange. For Ga/SiO_2, there were about 0.65 mol HD/ mol Ga was formed (Supplementary Table 8), corresponding to 70% of the Ga sites on the catalyst at 250°C. At the same temperature, there were only 0.19 mol HD/mol Zn formed (Supplementary Table 8), corresponding to 19% of the Zn sites. This is consistent with the XAS results in Supplementary Table 7 showing that Ga/SiO_2 loses more Ga-O bonds than Zn/SiO_2 does Zn-O bonds and suggests that at 250°C, Ga^{3+} forms a larger concentration of Ga-H than Zn^{2+} does Zn-H. At higher

temperatures, the fraction of sites forming metal hydrides increases. For Ga/SiO_2 at 450°C, nearly 100% of the Ga sites formed a Ga-H (Supplementary Figure 8, Supplementary Table 9).

Fourier Transform Infrared Spectroscopy (FTIR). FTIR was performed on Ga/SiO_2 by 1) treatment at increasing temperature in pure H_2 up to 550°C and then subsequent exposure to pure C_2H_4 at 250°C and 2) treatment in pure C_2H_4 at increasing temperature up to 250°C. All IR spectra reported for the treated samples are difference spectra in which that of the dehydrated sample was subtracted (*i.e.* $Ga/SiO_{2\ treated} - Ga/SiO_{2\ dehydrated}$).

When Ga/SiO_2 was treated at increasing temperature in H_2, it was apparent that there was an increase in features at 3745 cm^{-1}, 2034 cm^{-1}, and 1875 cm^{-1} with increasing temperature. The feature at 3745 cm^{-1} corresponds to Si-OH vibrations while those at 2034 cm^{-1} and 1875 cm^{-1} have been reported to correspond to Ga-H. The heterolytic dissociation of H_2 would facilitate the formation of a hydride and proximal surface silanol group. The spectra do not show evidence of Bronsted acid vibrations (3670 cm^{-1}), consistent with the nonacidic nature of SiO_2. As the catalyst was cooled back to room temperature in H_2, the features disappeared, like with XAS. Subsequent exposure to C_2H_4 results in the formation of an increasing feature at 2955 cm^{-1} with temperature, corresponding to C-H vibrations.

When Ga/SiO_2 was treated directly in C_2H_4, the 2955 cm^{-1} grew in with temperature. However, there was also an increase in an additional peak at 2970 cm^{-1}, corresponding to a vinylic alkyl (Supplementary Figure 10). Because these are difference spectra, it can be interpreted that any changes are due to changes to Ga/SiO_2 rather than excess ethylene in the cell. This indicates that Ga/SiO_2 can heterolytically cleave C-H bonds without the H_2 pretreatment leading to formation of a Ga-H reaction intermediate.

Density Functional Theory (DFT). A model for single site Ga^{3+} ions in an amorphous silica was created by substitution of Si atoms by Ga atoms. To account for the local charge balance, a proton was added onto the oxygen atom adjacent to the Ga, resulting in a silanol group. Multiple such DFT optimizations were performed to develop the basis structures for modeling ethylene oligomerization on Ga/SiO_2.

During the optimizations, nearby Si-OH groups were generated. Two representative Ga sites, including a three-coordinated (3CN) and four-coordinated (4CN), were then evaluated (Supplementary Figure 12). The different coordination environments are attributed to the original local binding condition of the Si atoms substituted. For the 3CN site, the Si atom is in a less constrained framework, where the Si-O bonds were elongated (a=1.80 Å, b=1.66 Å, c=1.76 Å, and d=1.69 Å). The cleavage of the Si-O bond (a) to generate the 3CN Ga site led to Ga-O bond distances of 4.51 Å, 1.86 Å, 1.82 Å, and 1.86 Å. A bond distance of 4.51 Å is sufficiently far away that it does not interact with the 3CN Ga site. In contrast, the Si atom used for creating the 4CN site is in a more constrained environment containing shorter Si-O bonds (a=1.67 Å, b=1.65 Å, c=1.66 Å, and d=1.68 Å). Consequently, the Si-OH formed on the 4CN Ga site is nearer to the metal center, and there are 4 Ga-O bonds at 2.62 Å, 1.84 Å, 1.83 Å, and 1.87 Å. The average Ga-O bond distance of the 4CN Ga site is 2.04 Å, which agrees with the experimental characterization (XAS).

Ga-H is considered as a key intermediate in facilitating olefin oligomerization. The free energy landscape of Ga-H formation, and the subsequent ethylene oligomerization on the Ga sites containing different geometries, are outlined in Supplementary Figures 13-15 and Supplementary Tables 11-13. The heterolytic cleavage of the C(sp^2)-H bond in ethylene results in a loss of a Ga-O bond and the formation of both a vinylic alkyl containing a C atom (bearing a negative charge) and a neighboring hydroxyl group.

A subsequent ethylene can then insert into the Ga-vinyl intermediate. A migratory insertion reaction leads to the Ga-butenyl species. β-H elimination causes butadiene to desorb from the metal center, thus forming a Ga-H intermediate. Here, the migratory insertion of ethylene has an intrinsic activation barrier of 1.45 eV. β -H elimination has an intrinsic activation barrier of 2.07 eV, which is a similar magnitude to energy barriers previously reported for Zn/SiO_2 for elementary steps with a similar geometry of transition states for propane dehydrogenation.[3] Once the Ga-H site is formed, it can facilitate subsequent oligomerization.

While the experimental evidence suggests that 4CN Ga sites are prominent in the pre-catalyst, it is possible to have a small concentration of sites that are less constrained in the silica framework (for example, in a 3CN Ga site). Because of the local environment on the SiO_2 framework, the Ga center is more geometrically available to bind with C_2H_4 (a less constrained environment). Therefore, lower reaction energetics following the same energy landscape are possible on the Ga-H formed using the less constrained site. The lower strain conditions reduce the intrinsic barrier of the C_2H_4 insertion step by 0.20 eV (Supplementary Figure 15, Supplementary Table 13). Because the Ga site may locate in a wide range of strain conditions in the amorphous silica, it is possible that a small concentration of less constrained Ga sites exist in the catalyst and could contribute to the catalytic activity.

Harmonic vibrational state analysis formed as the basis for estimating entropies of the adsorbates. However, for vibrational modes with low wavenumbers (< 150 cm^{-1}), particle-in-a-box (PIB) and free rotor schemes were used for calculating their contributions to the entropies. For the low frequency modes that resemble translations of a molecule, the PIB model is used with a length scale corresponding to the size of the cavity where the Ga site is located; this approximation should produce a lower limit of the entropy of these modes. For low frequency modes that resemble rotations, the free rotor approximation is used and likely represents a small overestimate of the corresponding entropies. We note that, for all intermediates in the oligomerization cycle, two modes representing the vibrations of the Ga site are found below 150 cm^{-1}. However, we do not consider their contributions to the entropy since the same vibrations (± 13.5 cm^{-1}) are found for bare Ga-H sites, and hence the entropy contributions will cancel. Finally, the entropies of the transition states are approximated as being equivalent to the entropies of the corresponding reactants or products, depending upon whether the geometry of the transition state more closely resembles that of reactants or products. Supplementary Table 14 outlines examples of entropy calculations for Ga-n-butyl and Ga-ethyl intermediates.

Supplementary Discussion

Single site Ga/SiO_2 and Zn/SiO_2 have been previously shown to be active for propane dehydrogenation and propylene hydrogenation reactions.[2,3] These reactions are proposed to have similar elementary steps as required for olefin oligomerization; however, different reaction conditions are required.

Similarities of Alkane Dehydrogenation, Olefin Hydrogenation and Oligomerization Reactions on Single Site Main Group and Post Transition Metal Catalysts

Ga/SiO_2 and Zn/SiO_2 have the same 4-coordinate (Td) structure as Ni/SiO_2 and are stable at high temperature, even in the presence of H_2. Whereas, Ni^{2+} reduces to Ni^0 at temperatures higher than 350°C in reducing conditions, losing oligomerization activity.[4] This work shows that Ga/SiO_2 and Zn/SiO_2 perform oligomerization with varying product distributions and the catalytic performance was investigated through a mechanistic understanding of the elementary steps and reaction intermediates required for oligomerization to occur. The ability to heterolytically activate C-H bonds is important for catalyst activation.[13–16] This capability is known through the previously reported dehydrogenation activity.[2,3]

In dehydrogenation (550°C, 1 atm), one C(sp3)-H bond is heterolytically cleaved across a M-O bond to form a metal-alkyl and a surface Si-OH (Supplementary Figure 16). Subsequent β-hydride elimination produces the olefin and forms a metal hydride intermediate. In hydrogenation (200°C, 1 atm), the microscopic reverse of dehydrogenation that occurs at lower reaction temperatures, the single site catalyst can activate H_2 to form a metal hydride. Olefin insertion to the metal hydride forms a metal alkyl which produces a paraffin when it is desorbed from the metal surface, reforming the initial catalyst, thus restarting the catalytic cycle.

Oligomerization occurs at 250°C, where the cleavage of the C(sp2)-H bond of ethylene occurs. The C(sp2)-H bond of ethylene is typically easier to cleave than the C(sp3)-H bond of propane because the resulting C(sp2)-M is more stable than the latter C(sp3)-M. IR demonstrates that higher reaction temperatures increase the C-H bond activation forming metal alkyls. For instance, at 250°C, the intensity of the C-H vibrations is notable, consistent with the oligomerization reactivity observed at that temperature on both Ga/SiO_2 and Zn/SiO_2.

From this, it is interpreted that hydrogenation/dehydrogenation (Supplementary Figure 16) and the proposed oligomerization reactions on main group single site catalysts (Figure 5) are related through the formation of the same reaction intermediates and the ability to perform the same elementary reaction steps. However, different reaction conditions are required. While dehydrogenation and hydrogenation can be performed at atmospheric pressure with high rates, oligomerization requires higher pressure to obtain high conversion and reasonable rates.

Understanding the reaction intermediates and elementary steps is critical for identifying novel oligomerization catalysts with tunable product selectivity for targeted applications. Ga/SiO_2 and Zn/SiO_2 are not traditionally used for olefin oligomerization. Though reports on Ga/ZSM-5 suggest the possibility of oligomerization on Ga ions, the acidic character of the support complicates the understanding of the metal activity.[17,18] ZSM-5 will protonate ethylene at room temperature and perform oligomerization at higher temperature. The lack of linear olefins and

observation of high Bronsted activity suggest that the acid character dominates performance on Ga/ZSM-5. Whereas the SiO_2 support in our study is nonreactive and the activity comes only from Ga^{3+} and Zn^{2+} sites respectively, thus forming linear olefins.

Supplementary References

1. Comas-Vives, A. Amorphous SiO2 surface models: energetics of the dehydroxylation process, strain, ab initio atomistic thermodynamics and IR spectroscopic signatures. *Phys. Chem. Chem. Phys.* **18**, 7475–7482 (2016).

2. Cybulskis, V. J., Pradhan, S. U., Lovon-Quintana, J. J., Hock, A. S., Hu, B., Zhang, G., Delgass, W. N., Ribeiro, F. H. & Miller, J.T. The nature of the isolated gallium active center for propane dehydrogenation on Ga/SiO_2. *Catal Lett.* **147**, 1252–1262 (2017).

3. Schweitzer, N. M., Hu, B., Das, U., Kim, H., Greeley, J., Curtiss, L. A., Stair, P. C., Miller, J. T. & Hock, A. S. Propylene hydrogenation and propane dehydrogenation by a single-site Zn2+ on silica catalyst. *ACS Catal.* **4**, 1091–1098 (2014).

4. Zhang, G., Yang, C. & Miller, J. T. Tetrahedral nickel(II) phosphosilicate single-site selective propane dehydrogenation catalyst. *ChemCatChem*. **10**, 961–964 (2017).

5. Joshi, R.m Zhang, G., Miller, J. T. & Gounder, R. Evidence for the coordination–insertion mechanism of ethene dimerization at nickel cations exchanged onto beta molecular sieves. *ACS Catal.* **8**, 11407–11422 (2018).

6. Brogaard, R. Y. & Olsbye, U. Ethene oligomerization in Ni-containing zeolites: theoretical discrimination of reaction mechanisms. *ACS Catal.* **6**, 1205–1214 (2016).

7. Phadke, N. M., Van der Mynsbrugge, J., Mansoor, E., Getsoian, A. B.; Head-Cordon, M. & Bell, A.T. Characterization of isolated Ga3+ cations in Ga/H-MFI prepared by vapor-phase exchange of H-MFI zeolite with GaCl3. *ACS Catal.* **8**, 6106–6126 (2018).

8. Getsoian, A .B., Das, U., Camacho-Bunquin, J., Zhang, G., Gallagher, J. R., Hu, B., Cheah, S., Schaidle, J. A., Ruddy, D. A.; Hensley, J. E., Krause, T. R., Curtiss, L. A., Miller, J. T. & Hock, A. S. Organometallic model complexes elucidate the active gallium species in alkane dehydrogenation catalysts based on ligand effects in Ga K-edge XANES. *Catal. Sci. Technol.* **6**, 6339–6353 (2016).

9. Geller, S. Crystal structure of β-Ga2O3. *J. Chem. Phys.* **33**, 676–684 (1960).

10. Britovsek, G. J. P., Malinowski, R., McGuinness, D. S., Nobbs, J. D., Tomov, A. K., Wadsley, A. W. & Young, C.T. Ethylene oligomerization beyond Schulz−Flory distributions. *ACS Catal.* **4**, 6922-6925 (2015).

11. Martínez, A., Arribas, M. A., Concepción, P. & Moussa, S. New bifunctional Ni–H-beta catalysts for the heterogeneous oligomerization of ethylene. *Appl. Catal. A: Gen*. **467**, 509–518 (2013).

12. Schreiber, M. W., Plaisance, C. P., Baumgartl, M., Reuter, K., Jentys, A., Bermejo-Deval, R. & Lercher, J. A. Lewis–Brønsted acid pairs in Ga/H-ZSM-5 to catalyze dehydrogenation of light alkanes. *J. Am. Chem. Soc.* **140**, 4849–4859 (2018).

13. Pidko, E. A., Hensen, E. J. M. & van Santen, R. A. Anionic oligomerization of ethylene over Ga/ZSM-5 zeolite: a theoretical study. *J. Phys. Chem. C*. **112**, 19604–19611 (2008).

14. Conley, M. P., Delley, M. F., Núñez-Zarur, F., Comas-Vives, A. & Copéret, C. Heterolytic activation of C-H bonds on Cr(III)-O surface sites ss a key step in catalytic polymerization of ethylene and dehydrogenation of propane. *Inorg Chem*. **54**, 5065–5078 (2015).

15. Delley, M. F., Silaghi, M. C., Nunez-Zarur, F., Kovtunov, K. V., Salnikov, O. G., Estes, D. P., Koptyug, I. V., Comas-Vives, A. & Copéret, C. X–H bond activation on Cr(III),O sites (X = R, H): key steps in dehydrogenation and hydrogenation processes. *Organometallics*. **36**, 234–244 (2017).

16. Copéret, C., Comas-Vives, A., Conley, M., Estes, D. P., Fedorov, A., Mougel, V., Nagae, H., Nunez-Zarur, F. & Zhizhko, P.A. Surface organometallic and coordination chemistry toward single-site heterogeneous catalysts: strategies, methods, structures, and activities. *Chem. Rev.* **116**, 323–421 (2016).

17. Kuz´min, I. V., Sokolova, N. A., Subbotina, I. R. & Zhidomirov, G. M. Ethylene adsorption and transformation on zeolite Ga+/ZSM-5. *Russ Chem Bull*. **64**, 278–283 (2015).
